# Supplementary material for: Characterizing multidimensional poverty in Migori County, Kenya and its association with depression
Source: PLoS One. 2021 Nov 16;16(11):e0259848. doi: 10.1371/journal.pone.0259848 (PMC8594838; doi:10.1371/journal.pone.0259848)
Supplement: S1 Table — Sensitivity analyses using continuous and ordinal outcomes in place of binary. (DOCX) [file pone.0259848.s002.docx]

**S2 Tables. Supplemental tables.**

| **Table A: Association with Multidimensional Poverty as Continuous** | | | |  |
| --- | --- | --- | --- | --- |
|  | Coefficient | 95% CI | p-value | |
| PHQ Depression Score | 0.272 | (0.169, 0.375) | <0.001 | |
| Age | 0.076 | (0.028, 0.125) | 0.002 | |
| Household Size | 0.452 | (0.134, 0.771) | 0.005 | |

Table A shows regression results using HDS as a linear variable scaled to 100. Regression is stratified by region and adjusted for marital status and income source.

**Table B: Poverty Status and PHQ-8 Depressive Symptoms**

| **Household Deprivation Score** | **OR** | **95% CI** | **p-value** |
| --- | --- | --- | --- |
| Non-poor (0-20% deprived) | Ref. | -- | -- |
| Vulnerable (21-33.2% deprived) | 1.115 | (0.945, 1.315) | 0.197 |
| Poor (33.3-50% deprived) | 1.608 | (1.277, 2.025) | <0.001 |
| Severely Poor (>50% deprived) | 1.837 | (1.059, 3.189) | 0.031 |

Table B shows regression results using PHQ-8 as an ordinal variable. Regression is stratified by region and adjusted for age, sex, and education.
