# Supplementary material for: Characterizing multidimensional poverty in Migori County, Kenya and its association with depression
Source: PLoS One. 2021 Nov 16;16(11):e0259848. doi: 10.1371/journal.pone.0259848 (PMC8594838; doi:10.1371/journal.pone.0259848)
Supplement: Attachment — Submitted filename: Response to Reviewers.docx [file pone.0259848.s004.docx]

**PONE-D-21-08861**

Dear Editors,

We very much appreciate this thoughtful feedback on our submission and are happy to make modifications accordingly. We have copied the reviewers’ comments below in bold and have given our responses, including any changes made to the manuscript, below each comment. We have also included both a version with tracked changes and a clean version of our manuscript with this submission.

**Reviewer Comments**

**Reviewer #1**

**I am glad to confirm that most of my comments are incorporated into the current manuscript.**

**However, theoretical backgrounds still need further explanation. There are many studies that established the association between poverty and depression. Then, why is it expected that MPI has a stronger association with depression than monetary wealth? What is the difference in the causal mechanism between MPI and monetary poverty?**

The theoretical background for this association is similar to the theoretical background for the multidimensional poverty index itself. An extensive discussion of this theoretical background is available through the OPHI (<https://ophi.org.uk/multidimensional-poverty-measurement-and-analysis-chapter-1/>). Simple income measures do not capture true deprivation, which is generally how those living in poverty conceive of their state. These deprivations are also the target of poverty mitigation policies and programs, not simply increased monetary income if this does not translate to less deprivation. Similarly, we believe a measure that actually captures deprivation—which is what is likely to affect mental state—more accurately captures the relationship with depression than a monetary measure which may or may not correlate with deprivation. It is also important to note the seasonality and unpredictability of monetary income in our setting (and similar settings), which makes the implementation of a unidimensional measure of monetary income very difficult and even less reliable. We have added further text to the Introduction to reflect the above.

**The contribution comparing to the previous study is also not clear. Especially, the authors mention that Callander et al. (2013) investigated the same issue in the Australian context. Then, what is the advantage of the authors’ study?**

The Callander study was performed in a high-income country, which is seen in the much lower rate of multidimensional poverty in their study. The rate was just 10% in the Australian context compared with nearly 20% in our low-income context in Kenya. Further, the Australian study utilized self-reported chronic illness or depression in place of our use of the PHQ-8. This is an important difference as many individuals, especially in low-resource settings, may not be formally diagnosed with depression. We feel that this expanded geographic and socioeconomic context as well as use of a validated screening tool allow us to build on the findings of Callander et al. We have added text to the Introduction to emphasize this.

**Reviewer #3**

**The Authors have tried to incorporate most of the comments and congratulations for that. However, high-quality English editing is required. The abstract and Introduction part still need to improve in writing before it got to publish. Interpretation of the tables can be improved.**

We have reviewed and edited the paper accordingly, resulting in several grammatical changes. We believe the paper to be written in grammatically correct English but welcome any specific grammar corrections. We have also added additional text to assist with table interpretation.

Thank you again for your consideration, and we look forward to hearing from you.

Sincerely,

Joseph R. Starnes
